# Recent Advances of Avian Viruses Research

**DOI:** 10.3390/v17010099

**Published:** 2025-01-14

**Authors:** Chi-Young Wang

**Affiliations:** Department of Veterinary Medicine, College of Veterinary Medicine, National Chung Hsing University, 250 Kuo Kuang Road, Taichung 40227, Taiwan; cyoungwang@dragon.nchu.edu.tw

The outbreaks of several epidemics caused by pathogenic avian viruses pose significant threats to the poultry industry. Nowadays, this problem also becomes an emergent issue to human health within the framework of One Health. Thus, more and more avian virologists have devoted themselves to exploring innovative approaches and strategies to study important pathogens, to gain the newest and valuable knowledge in order to assist in the control of those diseases. Therefore, several high-quality papers are assembled in this Special Issue.

The first paper in this Special Issue concerned avian influenza virus (AIV). The current AIV diagnostic process is to identify the virus via real-time reverse transcription–polymerase chain reaction (rRT-PCR). The virus is subsequently characterized by using whole-genome sequencing. This two-step diagnostic process takes days to weeks, but it can be expedited by using some novel sequencing technologies. The authors optimized a nucleic acid extraction setup from Oxford Nanopore Technologies for the rapid identification of AIV from clinical samples. The results showed that the magnetic-particle-based method was the most consistent regarding C_T_ value, purity, total yield, and AIV reads, and that it was less error-prone [1].

The second paper focused on the development of rapid, accurate, and cost-effective on-site diagnostic methods for the detection of avian leukosis virus (ALV) subgroups. The distinct trans-cleavage activity of Cas13, an RNA-guided RNA endonuclease, has been exploited in the molecular diagnosis of several viruses. The development and application of a Cas13a-based molecular test for the specific detection of the proviral DNA of ALV-A, B, and J subgroups were demonstrated. This novel system is based on the isothermal detection at 37 °C with a color-based lateral flow readout. The detection limit of the assay for ALV-A/B/J subgroups was 50 copies with no cross-reactivity with ALV-C/D/E subgroups and other avian oncogenic viruses, such as reticuloendotheliosis virus and Marek’s disease virus. It will benefit ALV detection in eradication programs [2].

The third paper explored whether the p17 protein of oncolytic avian reovirus (ARV) mediates cell migration and invadopodia formation. ARV p17 activates the p53/phosphatase and tensin homolog (PTEN) pathway to suppress focal adhesion kinase (FAK)/Src signaling and downstream signal molecules, thus inhibiting cell migration and the formation of invadopodia in murine melanoma cancer cell lines (B16-F10). It also suppresses the formation of the TKs5/NCK1 complex. This work provided new information about the p17-modulated suppression of invadopodia formation by activating the p53/PTEN pathway, suppressing the FAK/Src pathway, and inhibiting the formation of the TKs5/NCK1 complex [3].

The fourth paper in the Special Issue explored the prevalence of parrot bornavirus (PaBV) in Taiwan. Among 124 psittacine birds tested, 57 were PaBV-positive, a prevalence rate of 45.97%. Most of the PaBV infections were adult psittacine birds with a low survival rate (8.77%). A year of parrot bornavirus surveillance presented a seasonal pattern, with a peak infection rate in spring, indicating the occurrence of PaBV infections linked to seasonal factors. Severe meningoencephalitis and dilated cardiomyopathy in psittacine birds that suffered from proventricular dilatation disease were evident. PaBV-2 and PaBV-4 viral genotypes were found in the phylogenetic analyses [4].

The fifth paper in the Special Issue characterized the impact of infectious bronchitis virus (IBV) Delmarva/1639 and IBV Massachusetts 41 on chicken tracheal epithelial cells (cTECs) in vitro and the trachea in vivo. cTECs and young specific pathogen-free chickens were inoculated with IBV DMV/1639 or IBV Mass41, along with mock-inoculated controls, and the transcriptome was studied by using RNA-sequencing (RNA-seq) at 3 and 18 h post-infection for cTECs and at 4 and 11 days post-infection in the trachea. It was shown that IBV DMV/1639 and IBV Mass41 replicate in cTECs in vitro and in the trachea in vivo, inducing host mRNA expression profiles that are strain- and time-dependent. The different gene expression patterns between in vitro and in vivo tracheal IBV infection were demonstrated [5].

The sixth paper in the Special Issue characterized the impact of IBV Delmarva (DMV)/1639 and IBV Massachusetts (Mass) 41 on chicken tracheal epithelial cells (cTECs) and the trachea of chickens. cTECs and young specific pathogen-free chickens were inoculated with IBV DMV/1639 or IBV Mass41 and their transcriptomes were studied by using RNA-sequencing (RNA-seq) for cTECs and the trachea. It was shown that IBV DMV/1639 and IBV Mass41 replicate in cTECs and the trachea, inducing host mRNA expression profiles that were shown to be both strain- and time-dependent. The different gene expression patterns between in vitro and in vivo tracheal IBV infection were observed [6].

The seventh paper in the Special Issue studied interferon-inducible transmembrane protein 3 (IFITM3), which is an antiviral factor that plays an important role in the host innate immune response against viruses. In this study, the role of chicken IFITM3 in ARV infection was explored to show that this protein was localized in the cytoplasm. The homology analysis and phylogenetic tree analysis showed that the IFITM3 genes of different species exhibited great variation during genetic evolution, and chicken IFITM3 shared the highest homology with that of *Anas platyrhynchos* and displayed relatively low homology with those of birds such as *Anser cygnoides* and *Serinus canaria*. An analysis of the distribution of chicken IFITM3 in tissues and organs revealed that the IFITM3 gene was expressed at its highest level in the intestine and in large quantities in immune organs, such as the bursa of Fabricius, thymus, and spleen. Further studies showed that the overexpression of IFITM3 in chicken embryo fibroblasts (DF-1) could inhibit the replication of ARV, whereas the inhibition of IFITM3 expression in DF-1 cells promoted ARV replication. In addition, chicken IFITM3 exerted negative feedback regulatory effects on the expression of TBK1, IFN-γ, and IRF1 during ARV infection [7].

The eighth paper in the Special Issue examined how infectious bursal disease (IBD) is an immunosuppressive disease causing significant damage to the poultry industry worldwide. In Egypt, very virulent strains (such as genotype A3B2), responsible for typical IBD signs and lesions and high mortality, have historically prevailed. The present molecular survey, however, suggests that a major epidemiological shift might be occurring in the country. Out of twenty-four samples collected in twelve governorates in 2022–2023, seven tested positive for IBDV. Two of them were A3B2 strains related to other very virulent Egyptian isolates, whereas the remaining five were novel variant IBDVs (A2dB1b), reported for the first time outside of East and South Asia. This emerging genotype spawned a large-scale epidemic in China during the 2010s, characterized by subclinical IBD with severe bursal atrophy and immunosuppression. Its spread to Egypt is even more alarming considering that, contrary to circulating IBDVs, the protection conferred by available commercial vaccines appears suboptimal [8].

The ninth paper in the Special Issue combined transcriptome and translatome sequencing to investigate the mechanisms of transcriptional and translational regulation in the spleen after ARV infection. On a genome-wide scale, ARV infection can significantly reduce the translation efficiency (TE) of splenic genes. Differentially expressed translational efficiency genes (DTEGs) were identified, including 15 upregulated DTEGs and 396 downregulated DTEGs. These DTEGs were mainly enriched in immune regulation signaling pathways, which indicates that ARV infection reduces the innate immune response in the spleen. In addition, combined analyses revealed that the innate immune response involves the effects of transcriptional and translational regulation. Moreover, the key gene IL4I1 was discovered, which was the most significantly upregulated gene at both the transcriptional and translational levels. Further studies on DF1 cells showed that the overexpression of IL4I1 could inhibit the replication of ARV, while inhibiting the expression of endogenous IL4I1 with siRNA promoted the replication of ARV. The overexpression of IL4I1 significantly downregulated the mRNA expression of IFN-β, LGP2, TBK1, and NF-κB; however, the expression of these genes was significantly upregulated after the inhibition of IL4I1, suggesting that IL4I1 may be a negative feedback effect of innate immune signaling pathways. In addition, there may be an interaction between IL4I1 and ARV σA protein, and it was speculated that the IL4I1 protein plays a regulatory role by interacting with the σA protein. This study not only provided a new perspective on the regulatory mechanisms of the innate immune response after ARV infection but also enriched the knowledge of the host defense mechanisms against ARV invasion and outcomes of ARV evasion of a host’s innate immune response [9].

The tenth paper in the Special Issue compared IBV pathogenesis and host immune responses in young male and female chickens. One-week-old specific pathogen-free (SPF) White Leghorn male and female chickens were infected with Canadian Delmarva (DMV)/1639 IBV variant via the oculo-nasal route while maintaining uninfected controls, and these chickens were sampled at 4 and 11 days post-infection (dpi). No significant differences were observed between the infected male and female chickens in regard to IBV shedding, the IBV genome loads in all organs, and lesions in all tissues. The percentages of B lymphocytes were not significantly different between infected male and female chickens in all of the examined tissues. The percentages of CD8+ T cells were not significantly different between infected male and female chickens in all of the examined tissues, except in the trachea at 11 dpi, where female chickens had higher recruitment when compared with male chickens. Overall, the sex of chickens did not play a significant role in the pathogenesis of IBV, and only marginal differences in viral replication and host responses were observed that suggested more severity in male chickens infected by IBV [10].

The final paper reviews the current understanding about the use of antiviral chemotherapeutics in both farm poultry and companion birds. Some antiviral drugs, repurposed drugs originally used as antiparasitic drugs, other substances exhibiting antiviral activity, and novel peptides were described. Despite some drugs without pharmacokinetic and safety data having already been widely used in daily practice, the possible direction of further research on these drugs remains to be highlighted [11].

In conclusion, this Special Issue offers appealing portraits of recent advances in avian virus research. Its contents, encompassing molecular virology, viral immunology, viral pathogenesis, epidemiology, and antivirals, indeed provide some state-of-the-art advances in this field that can contribute to sustaining the health of poultry and humans to ensure the safety of public health and a sufficient food supply for the community. It surely provides a stellar example that will result in more exciting studies on this area in the near future.
